# The Use of Insecticide-Treated Curtains for Control of* Aedes aegypti* and Dengue Virus Transmission in “Fraccionamiento” Style Houses in México

**DOI:** 10.1155/2018/4054501

**Published:** 2018-06-19

**Authors:** Maria A. Loroño-Pino, Ana Uitz-Mena, Claudia M. Carrillo-Solís, Rocío J. Zapata-Gil, Dora M. Camas-Tec, Lourdes G. Talavera-Aguilar, Rosa C. Cetina-Trejo, Luis F. Flores-Flores, María C. Puc-Tinal, Clara Caamal-Jiménez, Guadalupe Reyes-Solís, Julián E. García-Rejón, Karla Saavedra-Rodriguez, Lars Eisen, Thomas J. Keefe, William C. Black IV, Barry J. Beaty

**Affiliations:** ^1^Centro de Investigaciones Regionales Dr. Hideyo Noguchi, Universidad Autónoma de Yucatán, 97000 Mérida, YUC, Mexico; ^2^Arthropod-Borne and Infectious Disease Laboratory, Colorado State University, Department of Microbiology, Immunology, and Pathology, Fort Collins, CO 80521, USA; ^3^Department of Environmental Health and Radiological Health Sciences, Colorado State University, Fort Collins, CO 80523, USA

## Abstract

Dengue, chikungunya, yellow fever, and Zika viruses transmitted by* Aedes aegypti* mosquitoes are major public health threats in the tropical and subtropical world. In México, construction of large tracts of “fraccionamientos” high density housing to accommodate population growth and urbanization has provided fertile ground for* Ae. aegypti*-transmitted viruses. We investigated the utility of pyrethroid-treated window curtains to reduce both the abundance of* Ae. aegypti* and to prevent dengue virus (DENV) transmission in fraccionamiento housing. Windows and doors of fraccionamiento homes in urban/suburban areas, where* Ae. aegypti* pyrethroid resistance associated with the Ile1016 knock down resistance (kdr) mutation in the voltage gated sodium channel gene was high, and in rural areas, where kdr resistance was low, were fitted with either insecticide-treated curtains (ITCs) or non-treated curtains (NTCs). The homes were monitored for mosquito abundance and DENV infection. ITCs reduced the indoor abundance of* Ae. aegypti* and the number of DENV-infected mosquitoes in homes in rural but not in urban/suburban study sites. The presence of non-treated screens also was associated with reduced numbers of mosquitoes in homes. “Super-infested” homes, yielding more than 50 mosquitoes, including DENV-infected mosquitoes, provide a significant public health risk to occupants, visitors, and people in neighboring homes.

## 1. Introduction

*Aedes aegypti*-transmitted arboviruses have exploded in public health importance throughout the tropical world. In the Americas, the emergence of epidemic dengue and dengue hemorrhagic fever in the 1980s and 1990s and the explosive emergence of chikungunya in 2013 and Zika viruses in 2015 in the new world are testimony to the epidemic potential of* Ae. aegypti*-transmitted arboviruses [1–4]. Many factors have contributed to the public health threats of* Ae. aegypti*-transmitted arboviruses, including erosion of public infrastructure, lack of effective and sustainable vector control, population growth, rapid and unplanned urbanization, and globalization, with the rapid jet transport of arboviruses and vectors throughout the world [5, 6]. Population growth and urbanization have combined to provide an ideal environment for* Ae. aegypti* throughout the tropical world with abundant peridomestic larval development sites and access to human hosts for female mosquitoes in indoor environments. Concomitantly, there has been a dramatic increase in knock down resistance (kdr) to pyrethroid insecticides in* Ae. aegypti* associated with specific mutations in the voltage gated sodium channel gene (vgsc) throughout the tropical world. This will likely compromise control efforts for* Ae. aegypti*-transmitted arboviruses [7, 8].

*Ae. aegypti* is highly anthropophilic and endophilic in Mexico and most of the tropical world, [9–12]; thus disruption of virus transmission by the mosquito in the home (and other indoor environments) provides a unique opportunity to prevent transmission of* Ae. aegypti*-transmitted arboviruses [13]. Indoor residual spraying of DDT was a major contributor to the success of the campaigns conducted by countries in the Western Hemisphere and the Pan American Health Organization to control* Ae. aegypti* and prevent yellow fever from emerging from sylvatic cycles [14]. More recently, a number of strategies have been used to control* Ae. aegypti* in the home, and some have proven to be effective, at least in the short term. These include indoor space and residual spraying with different insecticides and formulations, use of volatile pyrethroids in the home, and the use of curtains treated with long-lasting insecticides [13, 15–21]. Initial control efforts using insecticide-treated curtains (ITCs) successfully reduced* Ae. aegypti* in treated homes [15, 17, 22], but more recent studies have not been as successful [23]. Two major confounders of ITC efficacy are the type of housing to be protected and the dramatic increase in pyrethroid resistance in* Ae. aegypti* throughout the tropical world [24]. Pyrethroid resistance appears to be chiefly associated with specific mutations in the vgsc gene. The most prevalent mutations are Ile1016 and Cys1534 [25, 26]. Pyrethroid resistance is typically greater in urban areas, where space spraying of insecticides is part of the public health vector control programs [3].

We previously investigated a “Casa Segura” approach for control of* Ae. aegypti* and DENV transmission in two areas of Mérida, México, using a consumer product experimental design [17]. Windows in paired houses were provided with either insecticide-treated curtains (ITCs) or non-treated curtains (NTCs), and the houses were monitored for both* Ae. aegypti*, DENV-infected* Ae. aegypti*, and human DENV infections. DENV transmission as determined by detection of DENV-infected* Ae. aegypti* was reduced in one of the two study areas, in fact, dramatically so in homes where curtain usage was optimal. One possible reason for the differences in efficacy of the Casa Segura approach in the two areas may have been attributable to the predominance of fraccionamiento style housing in the study area where the curtains were most effective, whereas some houses in the other area were not a standard fraccionamientos design. These houses were more rural and frequently had large patios and backyards, thus providing a great variety and number of larval development and mosquito resting sites. In addition, in the course of the original study, we noted a dramatic increase in pyrethroid resistance in mosquitoes in Mérida, which may have confounded the results. A similar dramatic increase in pyrethroid resistance has been noted in* Ae. aegypti* throughout most of México [8, 24, 25, 27]. The potential operational significance and public health importance of pyrethroid resistance are of great concern.

In many countries, fraccionamiento style housing has been used to accommodate housing needs resulting from population growth and immigration. In a fraccionamiento, the cement homes are typically of one or a few designs with a limited number of doors and windows of standard sizes. Fraccionamientos with densely packed housing, large human populations with abundant susceptible individuals, and frequently inadequate vector control provide an ideal nidus for* Ae. aegypti* and arbovirus transmission. The fraccionamientos also provide unique opportunities to investigate new approaches to control* Ae. aegypti* and arbovirus transmission.

In the current study, we used a randomized cluster design to investigate the potential for a Casa Segura approach to control* Ae. aegypti* and DENV transmission in fraccionamiento style housing in urban/suburban and rural sites in Mérida and surrounding towns in Yucatán State, México. These sites were selected based in part upon the frequencies of mutations in the vgsc gene in local* Ae. aegypti* collections. Windows in intervention and control homes were fitted with ITCs or NTCs, respectively, and all experimental homes also received non-treated door curtains to reduce ingress or egress of* Ae. aegypti* mosquitoes. Homes were monitored for mosquito presence and abundance indoors and peridomestically. DENV infections and the frequency of mutations in the vgsc gene in the vectors and recent human dengue infections in study participants were also monitored.

## 2. Materials and Methods

### 2.1. Study Approach, Environment, and Design

A randomized cluster design was used to determine the potential for a Casa Segura approach using insecticide-treated window curtains and non-treated door curtains to protect individual homes and clusters of homes from* Ae. aegypti* and dengue virus. The timeline of activities is provided in Table 1.

Briefly, candidate, cement fraccionamiento style homes were identified in potential study sites in or near Mérida and other locations in Yucatán, México (Supplemental Figure 1). Study sites were selected in part based upon the prevalence of the Ile1016 allele in the resident* Ae. aegypti* mosquitoes [28]. Briefly, Ile1016 frequency was greater in mosquitoes in and around Mérida and lower in outlying, more rural communities, thereby providing the opportunity to investigate the efficacy of the Casa Segura approach in areas with differing frequencies of vgsc mutations. Fraccionamientos were located in the cities of Mérida (population ~ 800,000), Maxcanú (west of Mérida and population ~ 13,000), Caucel (west of Mérida and population ~ 6,988), Motul (east of Mérida and population ~ 23,000), and Umán (northwest of Mérida and population ~ 40,000), from Yucatán, México (Supplemental Table 1, Supplemental Figure 1). Locating bona fide fraccionamiento style homes in rural areas was difficult, resulting in the small number of rural study sites.

Pairs of intervention and control clusters of homes were randomly selected in the study sites. This pairing strategy was used to minimize the effect of spatial variability of DENV transmission intensity on the study design. For each of these pairs, one cluster/group was randomly selected to receive ITCs (intervention homes) and the other cluster/group to receive NTCs (control homes). Clusters were separated by a minimum distance of 500 meters.

A total of 445 homes in 42 clusters were enrolled in the study (Supplemental Table 1). Clusters contained 10–14 intervention or control homes, which were separated by <50 meters. The average lot size was 160 m^2^ in urban/suburban areas and 184 m^2^ in rural areas. Homes in urban/suburban areas had an average of 5 rooms (living room, kitchen, bathroom, and two bedrooms) and an average of 5 persons (range = 2–12 persons). Homes in rural areas had an average of 4 rooms (living room, kitchen, bathroom, and one bedroom) and an average of 5 persons (range = 1–11 persons).

A power analysis revealed that a two-arm study design comprising 40 pairs of clusters of homes (20 clusters of at least 10 intervention homes fitted with ITCs and 20 paired clusters of at least 10 control homes fitted with NTCs and with 5 persons/home) and assuming both a reasonable effect size of 50% (e.g., reduction in human infections from 10% to 5%) and an intra-cluster correlation as low as 0.02 would have a power of at least 85% to detect a statistically significant (*p* ≤ 0.05) reduction in infection rates in ITC versus NTC homes per year. We also assumed that there would be no or minimal inter-cluster correlation because the study clusters were located minimally 500 m apart, which exceeds the typical flight range of* Ae. aegypti* [29] to minimize spill-over or community effects from ITC treatment to NTC control clusters of homes.

Candidate home owners were approached to enroll in the study by trained nurses who informed the family members of the intent and scope of the study using a prepared script and an informed consent form. If the family agreed to participate, all family members > 1 year of age provided consent and were enrolled in the study; parents signed the consent form for minors after they agreed to participate. Participants were told if they were receiving ITCs or NTCs for the windows. NTCs were used for all doors. The Colorado State University Institutional Review Board required, as part of the consent process, that the families be told whether or not the curtains to be installed contained the insecticide and that NTCs be used for door curtains in all homes to minimize human contact with the insecticide.

DENV infections in* Ae. aegypti* were monitored in the intervention and control homes and clusters during the study. Mosquitoes were monitored for frequency of the Ile1016 allele, which is recessive in conferring pyrethroid resistance; only Ile1016 homozygotes are pyrethroid resistant [25].

Human serum samples were collected in November 2013, and recent DENV infections were detected by IgM ELISA as described previously [17].

### 2.2. Data Collection and Management and Study Approval

Study data were collected and managed using REDCap (Research Electronic Data Capture) hosted at Colorado State University and Universidad Autónoma de Yucatán [30]. REDCap is a secure, web-based application designed to support data capture for research studies, providing (1) an intuitive interface for validated data entry; (2) audit trails for tracking data manipulation and export procedures; (3) automated export procedures for seamless data downloads to common statistical packages; and (4) procedures for importing data from external sources.

The study was approved by the Institutional Review Board of Colorado State University and the Bioethics Committee of Centro de Investigaciones Regionales Dr. Hideyo Noguchi, Universidad Autónoma de Yucatán.

### 2.3. Window and Door Curtain Installation and Monitoring

Following enrollment of families into the study, homes were equipped with ITCs or NTCs. The insecticide-treated curtain material (Olyset Plus containing 2% permethrin insecticide and 1% piperonyl butoxide (PBO) synergist) and non-treated material were kindly provided by Sumitomo Chemical Corporation (Tokyo, Japan). Windows were treated with either ITCs (intervention homes) or NTCs (control homes) using the same curtain material used for ITCs; doors in both intervention and control homes were treated with NTCs. Sizes of windows and doors in each participating house were measured from February 1 to March 12, 2012. A total of 4,068 curtains were manufactured by 12 tailors from July to early September 2012. The average cost of each curtain including tailoring was approximately US $2.50. Curtains were installed in all windows and doors of 445 homes by late September to mid-October 2012. ITCs and NTCs were replaced in the study homes during August 2013 (Table 1).

### 2.4. Monitoring Homes and Clusters for Mosquitoes and Abundance

Adult mosquitoes were collected using CDC style backpack aspirators or BioGents Sentinel (BGS) traps (Biogents GmbH, Regensburg, Germany) [31, 32]. Backpack aspiration was used to monitor mosquito abundance inside study homes and outside on their patios or backyards at predetermined times (Table 1); this included two baseline collections prior to ITC/NTC implementation (September-October 2011 and August-September 2012) and 9 bi-monthly collections thereafter (November 2012 through June 2014). Collections included all rooms inside the home (indoors) and also on the patio (outdoors). The indoor collections included aspiration from furniture and behind hanging clothes and curtains. The length of time spent collecting per home varied by the number and size of rooms and the extent of the patio, but the overall time was based on previous work, typically in the range of 20 minutes [17].

Mosquitoes were also collected outside of study homes in the patio area using BGS traps on a predetermined schedule. These collections complemented those made by backpack aspiration around the homes and provided results to determine the surveillance potential of the two forms of mosquito collection. BGS traps were battery operated, equipped with the BG Lure, and set for a 24-hour period. BGS trapping of adults mosquitoes began October 2012 and finished in May 2014. Backpack aspiration and BGS trapping of specific sites were alternated in study sites (Table 1).

Field interdisciplinary teams comprised of 2 anthropologists, 1 nurse, and 6 entomologists also gathered information about potential larval development sites around the study clusters including total number of storm water drains (presence of water, size), vacant lots, abandoned houses, cemeteries, tire dumps, and other potential larval development sites.

### 2.5. DENV Detection in* Ae. aegypti* Females

After collection on a predetermined schedule (Table 1) by backpack aspiration or by BG Sentinel traps, mosquitoes were transported to the insectary in coolers for species identification using stereo microscopes and identification keys [33]. RNA and DNA were extracted for DENV detection [17] and Ile1016 genotype determination [28]. A cold chain was maintained from mosquito collection and transport to the laboratory and throughout the species identification and DENV detection processes.

*Ae. aegypti* females were assayed by RT-PCR for the presence of DENV RNA as described previously [9, 17]. Females were triturated individually and then a portion of the resulting sample was pooled, to contain up to 10 females, for RNA extraction using Trizol LS Reagent and DENV detection by RT-PCR. The remaining supernatant of adult females was stored at −70°C for virus isolation in C6/36 cells if a positive RT-PCR result was obtained. For pools testing positive for DENV RNA, the individual females were re-processed by RT-PCR, thereby allowing determination of DENV infection prevalence based on positive individual mosquitoes. The testing included the entire mosquito specimen, and the results therefore should be interpreted as a positive mosquito being infected with DENV but having an unknown status with regard to infectiousness to humans. Finally, samples from individual mosquitoes that were RT-PCR positive for DENV RNA were processed for virus isolation in C6/36 cells [34]. A total of 10,572 female* Ae. aegypti* were tested by RT-PCR and 382 by virus isolation.

### 2.6. Molecular Assay for vgsc Mutations in* Ae. aegypti* Mosquitoes

Previous studies revealed a rapid rise in the Ile1016 allele in* Ae. aegypti *in Mérida and the rest of México [17, 24]. The presence of the kdr-conferring genotype could potentially increase the likelihood of* Ae. aegypti* surviving contact with the pyrethroid-treated window curtains when entering or exiting the home. The frequency of the Ile1016 allele was determined annually in* Ae. aegypti* (females and males) collected both indoors with backpack aspirators and outdoors by backpack aspirators and BGS traps. Detection of the 1016 genotype in* Ae. aegypti* followed that used in previous studies [17, 24, 25].

### 2.7. Knock Down and Killing Efficacy of ITCs

ITCs and samples of the ITCs were placed in south- and north-facing windows of study homes and samples of ITCs were placed in interior regions of homes in September 2012 (Trial A) and replaced in September 2013 (Trial B) (Table 1). Curtains were replaced in year 2 of the study to preclude any possibility of permethrin or synergist degradation compromising the efficacy of the curtains after 12 months of use. In both trials, ITC samples were assayed for permethrin and PBO concentration and killing efficacy for* Ae. aegypti *on a predetermined schedule. Permethrin and PBO concentrations were determined at Sumitomo in Japan. Knockdown and killing efficacy of the ITCs were determined at Colorado State University. A cylinder assay [35] using a susceptible* Ae. aegypti* strain (New Orleans) and a field resistant strain collected in Mérida (Vergel) was used to characterize the ITC efficacy. Briefly, five 3-4-day-old female mosquitoes were aspirated into each cylinder, and after 3 minutes of exposure time, mosquitoes were transferred to cardboard cups. The knock down and mortality rates were then determined at 1 h and 24 h after treatment, respectively. Approximately 50 mosquitoes from each collection site were used to test each ITC.

### 2.8. Door and Window Curtain Usage Index

In our previous Casa Segura study in Mérida, we determined that appropriate consumer usage of curtains was important for reducing DENV transmission by* Ae. aegypti* in the homes [17]. In many homes, curtains were not always used appropriately; often curtains were removed or tied up, thereby permitting ingress and egress of* Ae. aegypti*. To address this, the following curtain usage index (CUI) was developed, and door and window curtain usage was determined during each home visit by one of the interdisciplinary field teams. Specifically, CUI was defined as the number of optimally used curtains divided by the total number of curtains in the home. Optimal use of a study curtain was defined as the curtain being present and extended (covering the window) and not covered by another non-treated curtain (typically a privacy curtain), which would aid mosquitoes in entering or exiting homes. In this current study, the CUI was expanded to cover appropriate usage of the door curtains. We determined the proportions of (1) windows/doors with our curtain present and (2) windows/doors with our curtain present and used optimally (not tied up or hanging together with privacy curtains). We then classified each house visit on the basis of terciles of their CUI values for all house visits as low (<0.25), medium (0.25–0.60), or high (>0.60).

### 2.9. Mosquito Screens and Usage in Participant Homes

The hyperabundance of* Ae. aegypti* in study sites prompted some home owners to physically screen some windows and install some screen doors to prevent intrusion by* Ae. aegypti*. Screening of doors and windows in NTC and ITC homes was documented by the visiting interdisciplinary field team. The screens were either standard fiberglass or metal insect screens mounted in aluminum or wooden frames and inserted in or covering windows (especially bedrooms). Screen doors were also sometimes affixed to exterior doorways. After curtain installation, the field teams recorded whether the screens were present, their locations, and how the screens were used in conjunction with the curtains. If the homeowners added screens during the study, the field teams recorded the presence of the new screens. By the end of the study, five intervention homes and four control homes had screens in all windows and doors. Other homes had variable numbers of screens, as well as variable numbers of windows and doors. The percent of windows and doors with screens was compared between ITC and NTC homes within both the urban/suburban and rural areas of the study.

### 2.10. Statistical Analysis

Data on indoor counts of female* Ae. aegypti*, all* Ae aegypti*, and mosquitoes of all species were first statistically evaluated via repeated-measures mixed-model analysis of covariance (ANCOVA) with treatment (intervention or control), area (urban/suburban or rural), and time (visit) as fixed effects and with the corresponding mosquito count at the visit prior to treatment as a covariate via application of the MIXED procedure in SAS® (SAS Institute, Cary, NC). Home-clusters and homes within clusters and all interactions of fixed and random effects were treated as random effects. All analyses were based on log-transformed counts to meet the inherent ANCOVA assumptions of homoscedasticity and normality. Because of differences in results between the urban and rural areas, as evidenced by significant interactions involving the main effect for area, as well as differences in Ile1016 allele frequencies between the two study areas, all analyses were carried out separately for the data collected for rural versus suburban/urban homes. The effects of both window screens and the curtain use were evaluated by the addition of one or both of these factors to the ANCOVA model either as a covariate or a categorical fixed effect. The Kenward-Roger option was utilized to accommodate nonsignificant interactions between fixed and random effects, and the compound symmetry covariance structure for repeated-measures was found to be preferable to an autoregressive structure. All pair-wise comparisons of least-squares means between intervention and control homes were based on the Tukey-Kramer procedure in SAS®. Data on each binary endpoint (in particular, human DENV infection) were to be analogously analyzed via application of the GLIMMIX procedure in SAS® for repeated-measures mixed-model multiple logistic regression (MLR) analysis; however, the MLR analysis could not be carried out due to the low number of human DENV infections during the study.

Bayesian odds ratios and highest posterior density (HPD) intervals at 95% were calculated using WinBugs [36, 37]. The effects of clusters, indoor ITC versus NTC, outdoor ITC versus NTC, and indoor versus outdoor collections on Ile1016 homozygote frequencies were analyzed in three-way contingency tables using the *G*-test [38].

### 2.11. Detection of Recent DENV Infections in Study Participants

A blood sample was obtained from each participant in November 2013 to determine if there had been recent DENV infections in participants (Table 1). All blood samples were obtained by trained nurses through finger prick and heparinized micro-hematocrit tubes. Plasma was obtained by centrifugation and stored at −70°C until tested. An IgM-capture enzyme linked immunosorbent assay was used to detect DENV-specific IgM antibodies [17, 39].

## 3. Results

### 3.1. Species and Numbers of Mosquitoes Collected during the Study

A total of 30,448 mosquitoes were collected in and around fraccionamento homes during the study. The numbers and species of mosquitoes collected in homes by backpack aspiration, outside homes by backpack aspiration, and outside homes in the patios by BG Sentinel traps are presented in Supplemental Table 2.

The total and average number of mosquitoes per home visit and* Ae. aegypti* collected inside of intervention and control homes in urban/suburban and rural study sites are presented in Table 2. The average numbers of all mosquito species, male and female* Ae. aegypti*, and female* Ae. aegypti *collected per home NTC and ITC home visit were reduced from the respective baseline averages for each of the three groups of mosquitoes (Table 2). Interestingly, there were no differences in the average number of mosquitoes collected per home visit between urban/suburban NTC and ITC homes. In contrast, in rural areas the average number of mosquitoes per home visit was greater in NTC than in ITC homes for all three groups of mosquitoes (Table 2). For example, the average number of female* Ae. aegypti* mosquitoes collected per home visit in rural homes fitted with NTCs was more than 2-fold greater than in rural homes fitted with ITCs (2.5 and 1.1, respectively), whereas the average number of female* Ae. aegypti* mosquitoes collected per home visit was the same in urban/suburban homes fitted with either NTCs or ITCs (1.1 and 1.1, respectively) (Table 2). The lower numbers of* Ae. aegypti* females collected in rural homes fitted with ITCs suggest that the rural mosquitoes were more susceptible to the ITCs, consistent with the lower Ile1016 allele frequencies found in rural versus urban/suburban mosquitoes.

### 3.2. Curtain Usage for Windows and Doors of NTC and ICT Homes

The usage of ITCs and NTCs in windows and doors was determined based on 9 visits (Nov 2012 to June 2014; Table 1) after the installation of the curtains in the fall of 2012. Among the four combinations of homes by area and treatment group, window curtain usage ranged from 79% to 87% while optimal window curtain usage ranged from 36% to 51% with limited differences between homes fitted with NTCs versus homes fitted with ITCs (Table 3). The effect of the CUI on the presence of infected* Ae. aegypti* in NTC and ITC homes is addressed below. Door curtain usage ranged from 55% to 73% among the four combinations of homes by area and treatment group, while optimal curtain usage on doors ranged from 41% to 48% also with limited differences between homes fitted with NTCs versus homes fitted with ITCs (data not shown).

### 3.3. Presence of Screened Windows in Control and Intervention Homes

The presence of screens in NTC and ITC homes was also determined and monitored per visit (Table 3). The use of screens varied both among study homes and within homes over the nine visits from no screened windows to all windows being screened. In evaluating the potential confounding effect of window screens on mosquito abundance, the number of unscreened windows per home visit was grouped as 0 or 1, 2 to 4, and 5 or more. The percent of home visits with 5 or more unscreened windows was significantly (*p* < 0.01) greater in ITC study homes than in NTC study homes in the urban/suburban area (45.8% and 32.8%, respectively) but was significantly (*p* < 0.01) less in ITC study homes than in NTC study homes in the rural area (32.8% and 48.9%, respectively, Table 3). Correspondingly, the percent of home visits with none or only 1 unscreened window was less in ITC study homes than in NTC study homes in the urban/suburban area (21.4% and 35.1%, respectively) but greater ITC study homes than in NTC study homes in the in the rural area (28.7% and 14.3%, respectively, Table 3).

### 3.4. Mosquito Abundances per Visit inside Study Homes for Female* Ae. aegypti*, All* Ae. aegypti,* and All Species

As noted above, the differences in mosquito abundances between NTC and ITC homes in each area (urban/suburban or rural) were statistically evaluated via repeated-measures analysis of covariance (ANCOVA), not only with treatment and time (visits 1 to 9) as fixed effects, but also with the CUI and the number of unscreened windows either as a covariate or grouped as a fixed effect. As might be expected, the number of unscreened windows was found to have a significant effect on mosquito abundance and thus was included after grouping as 0 to 1, 2 to 4, and 5 or more, as a factor in the final ANCOVA model. On the other hand, curtain usage, summarized via the CUI, was not statistically significant either as a continuous covariate or as a categorized main effect after accounting for window screening and thus was not included in the final ANCOVA. As also to be expected, counts for female* Ae. aegypti* (Table 4), all* Ae. aegypti *mosquitoes (Supplemental Table 3), and all mosquitoes of all species (Supplemental Table 4) varied significantly (*p* ≤ 0.05) over the nine visits in both the rural and urban/suburban areas.

ITCs in homes in the rural area were found to have an effect on reducing mosquito abundance inside homes; specifically, the difference between ITC and NTC homes in inside mosquito counts, although not statistically significant when averaged over all nine visits, was statistically significant (*p* ≤ 0.05) at seasonal peaks in mosquito abundance. In particular, the geometric mean of female* Ae. aegypti* mosquito counts inside rural homes was significantly lower for ITC homes than for NTC homes at visit 4 (0.9 and 3.7, respectively) and was marginally significantly less (0.05 < *p* < 0.10) at visit 9 (0.9 and 2.7, respectively; Table 4). These visits coincided with peak mosquito abundance in the study sites. These differences between ITC and NTC homes, with the same levels of significance, were even larger for rural homes with 5 or more unscreened windows. No such significant differences in female* Ae. aegypti* mosquito counts were found in the urban/suburban area (Table 4). Even during visits to rural homes when mosquito abundance was low, there was a consistent trend in which NTC homes yielded almost twice as many female* Ae. aegypti* as ITC homes (Table 4) in each visit, suggesting a sustainable protective effect of the ITCs. These results for both the urban/suburban and rural areas were essentially the same for counts of all* Ae. aegypti* mosquitoes with the exception that the difference between ITC and NTC homes in the rural area was significant (*p* ≤ 0.05) at visit 9 (1.2 and 4.3, respectively), as well at visit 4 (1.3 and 4.8, respectively; Supplemental Table 3).

The results for the total count of mosquitoes of all species were generally similar to those for the counts of both female and all* Ae. aegypti* mosquitoes. In particular, the geometric mean of the total counts of mosquitoes of all species inside rural homes was significantly less (*p* ≤ 0.05) for ITC homes than for NTC homes at both visit 4 (2.4 and 6.0, respectively) and visit 9 (2.3 and 5.9, respectively) (Supplemental Table 4).

### 3.5. Dengue Virus Infections in* Ae. aegypti* Females Collected inside and outside of NTC and ITC Homes

Overall, 59 DENV-infected* Ae. aegypti* females were collected either inside or from the patio of study homes (Table 5) during the study. After curtain installation 20 was detected in urban/suburban study sites and 10 in rural study sites. DENV-1 was detected in 14 (23.7%) and DENV-2 in 45 (76.3%) of the infected mosquitoes.

DENV infection rates were reduced from baseline values in mosquitoes collected inside study homes. Notably, in contrast to urban/suburban homes, no DENV-infected mosquitoes were detected inside rural ITC homes (Table 5). Interestingly, DENV infection rates were greater in mosquitoes collected outside rural ITC and NTC homes.

To examine these issues further, we calculated Bayesian 95% highest posterior density (95% HPD) intervals for the odds ratios of the outside infection rate relative to the inside infection rate (Table 5). No significant effect was found with the exception that the odds ratio for NTC homes in rural areas was 14.2 (95% HPD: 1.7–654.3), and the odds ratio for ITC homes in urban/suburban and rural area combined was 4.9 (95% HPD: 1.3–26.9). The odds of infection of mosquitoes collected outside rural NTC houses was 14-fold greater than the odds of infection in mosquitoes inside NTC houses. The odds of infection of mosquitoes outside ITC houses was 4.9 fold greater than that inside ITC houses.

Additional odds ratio analyses results found few significant effects (Table 6). In particular, the odds of infected mosquitoes in the urban/suburban area before NTCs were installed were 3.6-fold higher than after NTC installation (95% CI: 1.1–19.2). For the urban/suburban and rural areas combined, the odds of infected mosquitoes were 4-fold greater before NTC installation (95% CI: 1.4–16.0) and 4.3-fold greater before ITC installation (95% CI: 1.3–22.5). Also, there was a 10-fold greater odd of infected mosquitoes outdoors after NTC installation in rural versus urban/suburban areas.

### 3.6. *Aedes aegypti* Abundance and Infection Rates in Clusters of ITC and NTC Homes

Overall abundance of* Ae aegypti *females was compared for mosquitoes collected inside and from the patios of the ITC/NTC cluster pairs in the urban/suburban study sites and in the rural study sites (Table 7). The mean abundance of* Ae. aegypti* collected inside homes was 78 in NTC clusters and 72 in ITC clusters of urban/suburban homes and was 151 in NTC clusters and 69 in ITC clusters of rural homes.* Ae. aegypti* abundance was reduced in ITC homes in the rural clusters, where the* Ae. aegypti* were more susceptible to the ITCs. The mean abundance of* Ae. aegypti *females collected outside homes was 62 in NTC clusters and 58 in ITC clusters of urban/suburban homes and was 66 in NTC clusters and 56 in ITC clusters in rural homes (Table 7). Interestingly, DENV infection rates were greater in mosquitoes collected outside for both NTC and ITC rural homes.

### 3.7. Presence of Super-Infested Homes in the Study Areas

As in our previous study, some homes contained very large numbers of mosquitoes [17]. Fourteen super-infested homes (>50 mosquitoes of all species) in urban/suburban study areas and 4 in rural study areas were detected before curtain installation (Table 8). Following curtain installation, 7 super-infested homes were detected in urban/suburban study sites and 3 in rural study sites. The average number of mosquitoes collected in these urban/suburban NTC and ITC homes was 158 and 108, respectively (Table 8). In rural sites, the NTC homes yielded an average of 312 and 130 mosquitoes in NTC and ITC homes, respectively. Several of these homes also contained very large numbers of* Culex *mosquitoes. The average number of mosquitoes collected in the super-infested homes per visit after ITC/NTC installation did not differ dramatically in urban/suburban homes (NTC = 18; ITC = 14). In contrast, the number of mosquitoes collected per visit in rural NTC homes (35) was much greater than in ITC homes (14) (Table 8). These results suggest that the Casa Segura approach can be used to reduce (50%) the prevalence of super-infested homes and the number of mosquitoes in these homes, which provide a major threat to nearby homes.

### 3.8. Human Infections with DENV

Serum samples were obtained from 998 participants and were assayed by IgM ELISA for presence of antibodies to DENV. The number of human DENV infections in the study period was low. No DENV infections were detected in rural study sites. In urban/suburban homes, 7 of 435 (1.6%) serum samples collected from participants residing in NTC homes were IgM positive and 2 of 420 (0.48%) samples obtained from participants residing in ITC homes were IgM positive. In urban/suburban households there was a 3.41 fold (0.65–33.86) greater number of DENV infections in humans in NTC than ITC homes. DENV was not very active in our fraccionamiento sites during the study, accounting for the low number of human infections. Nonetheless, the trend is that participants in ITC homes were less likely to be infected than those living in NTC homes.

### 3.9. Monitoring of Allele Ile1016 in* Ae. aegypti* in the Study Sites and in Mosquitoes Collected inside and outside of Homes (and DENV Infection Rates)

Pyrethroid resistance in mosquitoes in the study sites was monitored and compared with data from archived mosquito DNA from our previous studies in Mérida in 1999 and 2007. In 1999, 0 of 272 mosquitoes contained an Ile1016 allele. In 2007, 26 of 100 (26%) of the mosquitoes were homozygous (Ile/Ile) and pyrethroid resistant. In 2010, when potential study sites were being characterized, 62% of urban/suburban and 7% of rural mosquitoes were resistant (Table 9). In 2012 at the time of curtain installation, 58% of urban/suburban mosquitoes and 28% of the rural mosquitoes were resistant. At the end of the study in 2014, 54% of the urban/suburban mosquitoes and 26% of the rural mosquitoes were resistant. There was a significantly greater frequency of Ile1016 homozygotes in urban areas in each of the six years (Table 9).

To determine the potential effect of ITCs on Ile1016 allele frequencies, mosquitoes were collected inside and outside of study homes and genotyped (Table 10). In the urban/suburban clusters, 58% of the* Ae. aegypti* collected in NTC homes were homozygotes and 54% of those collected outside of these homes were homozygotes. In the ITC clusters, 56% of the* Ae. aegypti* collected in the homes were homozygotes and 57% of those collected outside the homes were homozygotes. In rural clusters, 33% of the* Ae. aegypti* collected in the NTC homes were homozygotes and 34% of those collected outside of these homes were homozygotes. In the ITC clusters, 27% of the mosquitoes collected inside were homozygotes and 21% of those collected outside were homozygotes (Table 10).

Ile1016 allele frequencies were compared in mosquitoes collected indoors and outdoors of NTC and ITC homes and between clusters using the* G*-test [38]. Overall, genotype frequencies and sample sizes differed significantly among clusters in four analyses (Supplemental Table 5). In urban/suburban clusters, genotype frequencies did not differ significantly between mosquitoes collected indoors in NTC and ITC homes (*p* = 0.0544), and homozygotes were detected more frequently (*p* = 0.0403) outside of ITC homes than NTC homes (Supplemental Table 5). In rural clusters, differences in genotype frequencies of mosquitoes collected indoors in NTC and ITC homes (33% versus 27%) were barely significant (*p* = 0.0494), but homozygotes were significantly greater in frequency (34% versus 21%) in mosquitoes collected outside of NTC homes (*p* < 0.0001, Supplemental Table 5). The reason for this is unknown. Overall, these results suggest that the presence of ITCs exerts little or no selection for increased kdr in the mosquitoes as reflected by an increase in homozygotes in homes equipped with ITCs.

### 3.10. Knock Down and Killing Efficacy of ITCs

**Figure 1:**
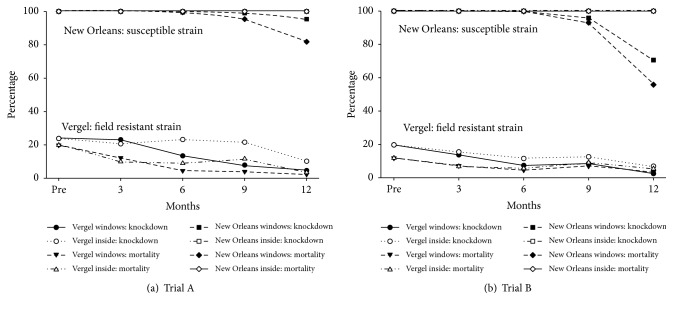
Curtain Toxicity Trials.

Knock down and killing efficacy of ITCs were determined in both years of the study using the susceptible (New Orleans strain) and a resistant Vergel strain of* Ae. aegypti.* In year 1 (Table 11, Figure 1, Trial A), almost 100% of the susceptible New Orleans strain of* Ae. aegypti* were killed following exposure to the ITCs (Table 11, Figure 1, Trial A); knock down and killing efficacy were reduced after 9 months if the ITCs had been hung in windows but not if the ITCs had been placed indoors (Table 11). The resistant Vergel strain of* Ae. aegypti* was much less susceptible to the ITCs at baseline (19.8%), and the knock down and killing efficacy of the ITCs declined over time with further reduction in killing efficacy after exposure in windows for 3 months (12.2% killed) and ≥6 months (<5% killed). In year 2 (Figure 1, Trial B), the results were almost identical to those obtained in Trial A (Figure 1).

### 3.11. Stability of Permethrin and PBO in the ITCs

The stability of permethrin and PBO in ITCs affixed to windows and in the home was measured at predetermined times during the study (Table 11, Supplemental Table 6). Permethrin remained relatively stable with only a 12% reduction in the active ingredient per kilogram of curtain in ITCs affixed to windows verses a 7% reduction in ITCs in the interior of the homes for the trial period (Supplemental Table 6). In contrast, PBO concentration was reduced 80% in ITCs affixed to windows versus 31% in ITCs from the interior of homes (Supplemental Table 6). The amount of permethrin and PBO in the ITCs was a function of both time and location in the home (Supplemental Table 6).

### 3.12. Comparison of Backpack Aspiration and BGS Traps for Surveillance of Mosquitoes outside of Study Homes

To determine which method would be most efficacious for surveillance of the principal arbovirus vectors in Mérida,* Ae. aegypti* (dengue, chikungunya, and Zika viruses) and* Cx. quinquefasciatus *(West Nile virus), we compared the number of mosquitoes collected by backpack aspiration and BGS traps from patios of urban/suburban and rural study sites (Table 12). In both urban/suburban and rural areas, the BGS traps collected significantly more* Ae. aegypti *and* Cx. quinquefasciatus* female mosquitoes than backpack aspiration. Backpack aspiration yielded more male* Ae. aegypti* in urban/suburban than in rural areas, presumably due to the closer presence of foliage to the fraccionamiento houses. Clearly, the use of BGS traps (if feasible) is superior to backpack aspiration for surveillance of females of these important disease vectors in outdoor environments. This may be due to the longer sampling time of the BGS traps, which were run over night, compared to the limited time and time of day devoted to backpack aspiration of patio areas.

## 4. Discussion

The emergence of pyrethroid resistance in* Ae. aegypti* throughout the tropical world is of great public health significance. The loss of this class of insecticides for control of* Ae. aegypti* and the arboviruses that it transmits (dengue, chikungunya, Zika, and yellow fever viruses) is a potential public health disaster [3]. As documented in our studies and by others, the increase in frequency of the kdr-conferring Ile1016 allele throughout México, even in rural areas, has been dramatic. Indeed, when we first surveyed potential study sites in 2009, kdr (Ile/Ile) frequencies ranged from 87% in mosquitoes collected from Vergel, 44% in Caucel, and 7% in Motul and Maxcanú. By the time that the curtains were installed, resistance rates in the large Mérida suburbs of Caucel and Umán had risen dramatically and were nearly equivalent to those in Mérida City. Even in the smaller towns, where public health spraying of insecticides is infrequent if at all, Ile1016/Ile1016 frequencies had risen to almost 30%.

In our previous study in Mérida [17] kdr allele frequencies were lower, and the effective use of ITCs reduced the presence of* Ae. aegypti* mosquitoes and DENV infections in humans in homes in one of the two study areas. In the current study, the presence of ITCs and NTCs and screens in homes exhibited protective efficacy in rural but not in urban/suburban homes. In rural homes, the mean number of* Ae. aegypti* collected per home visit (Table 2) was greater in NTC homes than in ITC homes. In rural areas, no infected mosquitoes were detected in ITC homes in contrast to NTC homes (Table 5). In contrast, the mean number of* Ae. aegypti* collected per home visit (Table 2) and DENV infection rate (Table 5) did not differ in urban/suburban sites. The number of mosquitoes and DENV infection rate were reduced in ITC clusters in rural but not in urban/suburban clusters (Table 7). Finally, the number of human DENV infections detected was 3.4-fold greater in NTC than ITC urban suburban homes, but the number of human infections was small. There were no human infections detected in rural study sites. In total, these data suggest that in the urban/suburban study sites, pyrethroid resistance may have compromised the protective effect of ITCs. Unlike our previous study [17], even urban/suburban homes with high CUI scores experienced significant* Ae. aegypti* infestation (Tables 2 and 4). The high kdr allele frequencies in mosquitoes in the urban/suburban fraccionamiento study sites (Table 9) likely compromised the protective efficacy of the curtains in the current study. Others have recently demonstrated that deltamethrin resistance has led to treatment failure for indoor control of* Ae. aegypti *in Mérida [27], which is consistent with our results.

The dramatic differences in knock down and killing efficacy of the ITCs for the susceptible New Orleans strain and the resistant Vergel strain of* Ae. aegypti* from Merida (Figure 1) are illustrative of how kdr likely compromised the protective efficacy of the ITCs. Similarly, the decline of the synergist (PBO) in ITCs, especially ITCs in windows (Table 11) overtime, could have contributed to the reduced protective efficacy of the ITCs by not impairing cytochrome P450 mediated pyrethroid resistance pathways in the urban/suburban mosquitoes [40]. Ominously, kdr allele frequencies are also increasing in* Ae. aegypti* in our rural study sites (Table 9), potentially limiting the use of pyrethroid-treated curtains in such areas. The dramatic increases in kdr in* Ae. aegypti* in México [25, 26] caused public health authorities to ban the use of permethrin (a pyrethroid used from 1998 to 2010 for ULV space spraying) and to approve the use of other insecticides to control* Ae. aegypti* [41, 42]. In Mérida, public health authorities replaced permethrin with phenothrin for ULV space spraying in 2011 and then switched to the organophosphate chlorpyrifos in 2012. Pyrethroid use continued for indoor residual spraying until it was eventually replaced by the carbamate propoxur. Clearly, development of new insecticides and effective control practices are critical to mitigate resistance and to increase the armamentarium for control* Ae. aegypti* and the viruses it transmits [3]. Development of new active ingredients, formulations, and/or insecticide combinations and environmentally stable formulations that can be delivered via curtains is critical for future Casa Segura approaches to control* Ae. aegypti.* Development of curtains treated with two insecticides with different modes of action [43] or electrostatically treated curtains/netting [44] for resistance breaking and control of resistant mosquitoes are exciting examples of possibilities in this arena.

Our results suggest a limited protective effect of the ITCs on* Ae. aegypti* abundance and DENV infection in mosquitoes in rural homes, but we feel that the potential protective efficacy of the curtains may be underestimated in this study for a number of reasons. For example, statistically significant reductions in female* Ae. aegypti* numbers were demonstrable in rural ITC versus NTC homes during periods of peak abundance (Visits 4 and 9, Table 4), but not in other visits. The statistical significance of the former and the non-significance of the latter are directly correlated with the abundance of female* Ae. aegypti* in these sampling periods. The numbers of mosquitoes collected in the limited number of rural fraccionamiento homes studied was simply too low to demonstrate statistically the protective effect. However, rural NTC homes invariably yielded almost twice as many* Ae. aegypti* females as ITC homes at non-peak abundance visits (Table 4). This protective trend was not observed in the urban/suburban homes (Table 4). It is also noteworthy that DENV activity was greatly reduced in our study areas during the two years of the intervention. This was more reminiscent of dengue endemicity than hyperendemicity, which makes demonstration of a protective effect of ITCs more difficult [23]. The low numbers of infected mosquitoes and humans complicated demonstration of a protective effect of ITCs (Tables 5 and 6). In this regard, in our previous study [17], the protective effect of the ITCs was demonstrable in one study area but not in the other area with reduced mosquito numbers and DENV activity.

Noncompliant usage of window and door curtains by study participants despite extensive training in their appropriate usage likely comprised the protective efficacy of the curtains in the rural area (Table 3). Such misusage would do little to stop ingress and egress of* Ae. aegypti.* More compliant window curtain usage would also likely have enhanced the protective effect; less than 50% of the curtains were used optimally (Table 3). Similarly, the appropriate usage of door curtains was less than 50%. More compliant usage of the door curtains may have improved the protective effect. It is also noteworthy that door curtains were not treated with insecticide, which may have increased the effectiveness of the curtains. Our free hanging ITC doors were problematic. Development of inexpensive, durable insecticide-treated screened doors would likely provide greater and longer lasting protection against* Ae. aegypti.* Several studies have now reported on the use of insecticide-treated screens that effectively seal doors and windows for better control of* Ae. aegypti* in México [45–48]. The presence of screens did help reduce* Ae. aegypti* abundance in homes in our study (Table 4). Such approaches may provide better protection against* Ae. aegypti *and DENV.

As in our previous study, the ITCs and NTCs were very well received by study participants [17]. ITCs can be an attractive and protective addition to the homes [19]. Insecticide-treated curtains or screens provide a safe and environmentally sensitive platform for mosquito control. Clearly, a market exists for ITCs for mosquito control [17]. Unique products combining ITCs and screens to maximize protection of and air circulation in homes may promote better usage and provide longer term protection against mosquitoes. The public health needs and opportunities for such new products are great.

In our previous studies in Mérida, we demonstrated the strong endophily of* Ae. aegypti *in homes and schools [9, 49].* Ae. aegypti* were also collected by backpack aspiration in patios and other peridomestic locations surrounding the homes, but despite using a standardized protocol, we were concerned that backpack aspiration may not have as efficiently collected* Ae. aegypti* in the peridomestic environment as in the indoor environment. BGS traps were used near study homes to augment collection of* Ae. aegypti* (Table 12). Significantly more* Ae. aegypti* females were collected in urban and rural areas by the BGS traps than by backpack aspiration. The increased collection efficiency also resulted in detection of more DENV-infected mosquitoes in the peridomestic environment (Table 7). To our surprise, the majority of infected mosquitoes, especially in the rural study sites, were collected peridomestically around both ITC and NTC homes, suggesting the potential for significant peridomestic DENV transmission. We do not feel that this is attributable to the presence of curtains driving infected mosquitoes into the peridomestic environment. Indeed, the increased DENV infection rate was greater in the peridomestic mosquitoes during the baseline studies (Table 7). It is also noteworthy that even when DENV-infected mosquitoes were detected in patios, infected mosquitoes were not detected in the first year after ITC installation inside homes in urban study sites or in rural study sites (data not shown). The potential epidemiologic significance of the infected mosquitoes in the peridomestic environment remains to be determined. Clearly however the use of BGS traps instead of backpack aspiration for* Ae. aegypti* surveillance in the peridomestic environment could be of great value for surveillance programs.

The presence of super-infested homes in the study sites remains of great concern (Tables 8(a) and 8(b)). Of the 445 homes that participated for the entire study, 18 (4%) yielded more than 50 mosquitoes and were designated as super-infested homes. Twelve of these were homes equipped with NTCs and 6 were homes equipped with ITCs (Table 8(a)). Following installation of curtains, the number was reduced to 10 super-infested homes; 7 of which were homes with NTCs and 3 with ITCs. The average number of mosquitoes collected per NTC and ITC home was 158 and 108, respectively, for urban/suburban homes and 312 and 130, respectively, for rural homes, suggesting a protective effect of the ITCs even in super-infested homes (Table 8(b)). Emphasizing the threat that super-infested homes pose, 7 DENV-infected* Ae. aegypti* were detected in one of the super-infested homes. The presence of these super-infested homes and their public health significance were discussed in our previous study [17]. Such homes are clear public health threats that need to be accounted for in vector control efforts.

## Figures and Tables

**Table 1:** Study schema: timeline of activities for the study.

Activities	2012				2013												2014						
	A	S	O	N	D	J	F	M	A	M	J	J	A	S	O	N	D	J	F	M	A	M	J
Recruitment- Basic data of families and homes	●	●																					
Informed consent	●	●																					
Mosquito surveillance – Backpack aspirations	●	●		●	●		●	●		●		●		●	●		●		●		●		●
Curtain installation			●																				
Mosquito surveillance – BG sentinel traps			●			●		●	●		●		●			●		●		●		●	
Curtain replacement													●										
Nurse contacts – febrile diseases, etc.				●	●		●	●		●		●		●	●		●		●		●		●
Curtain usage monitoring				●	●		●	●		●		●		●	●		●		●		●		●
Presence of screens in windows and doors				●	●		●	●		●		●		●	●		●		●		●		●
DENV-infected mosquitoes detected	●		●			●					●				●	●	●	●	●	●	●	●	●
Serologic survey of participants																●							

**Table 2:** Total number and average^1^ number per home visit of all species of mosquitoes and *Aedes aegypti* males and females collected inside intervention (ITC)^2^ and control (NTC)^2^ homes during pre- and post-curtain installation.

Area	NTC/ITC	Baseline 2012			Visits 1–9		
		Total number and average number per home of mosquitoes before curtain installation			Total number and average number per home visit of mosquitoes after curtain installation		
		All species	*Aedes aegypti* female and male	*Aedes aegypti* female	All species	*Aedes aegypti* female and male	*Aedes aegypti* female
Urban/Suburban	NTC	1,036 (6.2)	826 (5.0)	498 (3.0)	3,588 (2.9)	2,234 (1.8)	1,409 (1.1)
	ITC	793 (4.7)	618 (3.7)	383 (2.3)	3,126 (2.6)	2,088 (1.7)	1,301 (1.1)
Rural	NTC	392 (16.3)	143 (6.0)	72 (3.0)	1,537 (8.4)	651 (3.6)	452 (2.5)
	ITC	287 (11.0)	84 (3.2)	61 (2.3)	753 (3.9)	339 (1.7)	206 (1.1)

^1^Average is shown in parentheses; ^2^ITC = insecticide treated curtains; NTC = non-treated curtains.

**Table 3:** Window curtain and screen usage for intervention (ITC) and control (NTC) homes during the treatment period.

Area	Total number of home visits		Mean^1^ percent of windows with our curtains present		Mean^1^ percent of windows with our curtain present and used optimally		Percent^2^ with ≤1 unscreened windows		Percent^2^ with 2–4 unscreened windows		Percent^2^ with ≥5 unscreened windows	
	NTC	ITC	NTC	ITC	NTC	ITC	NTC	ITC	NTC	ITC	NTC	ITC
Urban/Suburban	1,258	1,203	79.2	81.6	50	50.5	35.1	21.4	19.1	20.5	45.8	58.1
Rural	182	195	86.9	83.5	43.1	35.6	14.3	28.7	36.8	38.5	48.9	32.8

^1^Least-squares mean from repeated-measures ANCOVA of the percent of homes with curtains present or present and used optimally with the number of unscreened windows as a covariate; NTC and ITC homes were not significantly different in either area (*p* > 0.20); ^2^NTC and ITC homes in both areas were significantly different in the percent with ≤1 and percent with ≥5 unscreened windows (*p* < 0.01) but not in the percent with 2–4 unscreened windows (*p* > 0.35).

**Table 4:** Geometric mean^1^ count for female *Aedes aegypti* mosquitoes in fraccionamiento homes by visit number, area, curtain treatment group, and number of unscreened windows in control (NTC) and intervention (ITC) homes.

Area	Number of		Visit									Overall
	Unscreened Windows	Treatment	1	2	3	4	5	6	7	8	9	
	≤1	NTC	0.56	0.30	0.39	0.72	0.52	**0.46**	0.31	0.18	0.28	0.40
		ITC	0.27	0.17	0.34	0.70	0.57	**0.95**	0.59	0.22	0.42	0.45
	2 to 4	NTC	0.64	0.12	0.35	0.77	0.83	0.75	0.38	0.19	0.63	0.50
Urban/suburban		ITC	0.57	0.36	0.38	0.70	1.12	1.14	0.43	0.22	0.48	0.57
	≥5	NTC	0.38	0.21	0.53	1.11	0.99	0.77	0.55	0.12	0.86	0.58
		ITC	0.47	0.26	0.30	1.37	1.15	0.77	0.89	0.11	0.61	0.61
	Overall	NTC	0.53	0.21	0.42	0.86	0.77	0.65	0.41	0.16	0.57	0.49
		ITC	0.43	0.26	0.34	0.90	0.93	0.95	0.62	0.18	0.50	0.54

	≤1	NTC	0.50	0.58	0.55	**3.43**	1.19	0.93	1.19	0.56	1.26	1.01
		ITC	0.32	0.12	0.41	**0.26**	0.53	0.52	0.48	0.18	0.11	0.32
	2 to 4	NTC	0.50	0.79	0.33	3.56	1.97	1.10	0.43	0.21	3.25	1.09
Rural		ITC	0.11	0.19	0.81	1.72	0.73	1.20	0.57	0.23	1.55	0.71
	≥5	NTC	0.52	1.40	0.32	**4.07**	2.42	**2.88**	1.35	0.34	**4.06**	1.59
		ITC	0.52	0.49	0.10	**1.07**	1.96	**0.71**	0.98	0.10	**1.23**	0.71
	Overall	NTC	0.51	0.90	0.40	**3.68**	1.81	1.51	0.95	0.36	**2.65**	1.21
		ITC	0.30	0.26	0.41	**0.92**	0.99	0.79	0.66	0.17	**0.85**	0.57

^1^Geometric means are significantly different between the control (NTC) and intervention (ITC) treatment group of homes at the 10% significance level if bolded and at the 5% significance level if bolded and underlined.

**Table 5:** Dengue virus infections in *Aedes aegypti* females collected inside and outside of control (NTC) and intervention (ITC) homes from Aug. 2012 to June 2014.

Area	City	BEFORE CURTAIN INSTALLATION DENV+/total tested		AFTER CURTAIN INSTALLATION DENV+/total tested			
				NTC		ITC	
		Inside (BP^1^)	Outside (BP)	Inside (BP)	Outside (BP and BG^2^)	Inside (BP)	Outside (BP and BG)
Urban/suburban	Mérida	3/1,987	1/371	1/1,058	4/1,009	3/1,079	4/913
	Umán	15/440	4/39	1/301	0/180	0/117	6/205
	Caucel	1/25	0/0	1/42	0/25	0/94	0/46
	*Total*	*19/2,452*	*5/410*	*3/1,401*	*4/1,214*	*3/1,290*	*10/1,164*
	*Infection Rate per 1,000 mosquitoes*	*7.7*	*12.2*	*2.1*	*3.3*	*2.3*	*8.6*

	*Odds Ratio (95% HPD)*	1.58 (0.46–4.41)		1.54 (0.26–10.54)		3.72 (0.95–21.06)	

Rural	Maxcanú	0/30	0/4	1/181	3/72	0/26	1/23
	Motul	4/194	1/13	0/271	3/124	0/180	2/143
	*Total*	*4/224*	*1/17*	*1/452*	*6/196*	*0/206*	*3/166*
	*Infection Rate per 1,000 mosquitoes*	*17.9*	*58.8*	*2.2*	*30.6*	*0.0*	*18.1*

	*Odds Ratio (95% HPD)*	3.41 (0.03–15.21)		*14.19 (1.70–654.28)*		NA	

Total	*Urban/Suburban and Rural*	*23/2,676*	*6/427*	*4/1,853*	*10/1,410*	*3/1,496*	*13/1,330*
	*Infection Rate per 1,000 mosquitoes*	*8.6*	*14.1*	*2.2*	*7.1*	*2.0*	*9.8*
	*Odds Ratio (95% HPD)*	1.64 (0.54–4.18)		3.30 (0.950–14.44)		*4.91 (1.34–26.92)*	

^1^Mosquitoes collected by backpack aspiration; ^2^Mosquitoes collected by backpack aspiration and BGS traps outside of homes.

**Table 6:** Bayesian 95% highest posterior density (HPD) intervals for the odds ratios^1^ of mosquito infection rates in control (NTC) and intervention (ITC) homes.

(A) Urban/Suburban			

Comparison	Odds ratio	lower 95% HPD	upper 95% HPD
(1) Inside (Before) vs Inside (After – NTC)	**3.64**	**1.07**	**19.23**
(2) Inside (Before) vs Inside (After – ITC)	3.35	0.98	17.70
(3) Outside (Before) vs Outside (After – NTC)	3.73	0.80	18.89
(4) Outside (Before) vs Outside (After – ITC)	1.42	0.38	4.61
(5) Outside (Before) vs Periphery	2.31	0.38	24.42

(B) Rural			

Comparison	Odds ratio	lower 95% HPD	upper 95% HPD
(1) Inside (Before) vs Inside (After – NTC)	8.17	0.80	403.82
(2) Inside (Before) vs Inside (After – ITC)	0.00	0.00	1.64
(3) Outside (Before) vs Outside (After – NTC)	1.97	0.04	17.90
(4) Outside (Before) vs Outside (After – ITC)	3.36	0.06	44.75
(5) Outside (Before) vs Periphery	3.39	0.07	37.12

(C) Total			

Comparison	Odds ratio	lower 95% HPD	upper 95% HPD
(1) Inside (Before) vs Inside (After – NTC)	**4.01**	**1.37**	**15.97**
(2) Inside (Before) vs Inside (After – ITC)	**4.31**	**1.30**	**22.48**
(3) Outside (Before) vs Outside (After – NTC)	1.99	0.59	6.10
(4) Outside (Before) vs Outside (After – ITC)	1.44	0.45	4.10
(5) Outside (Before) vs Periphery	1.41	0.37	5.32

(D) Urban vs Rural			

Comparison	Odds ratio	lower 95% HPD	upper 95% HPD
Urban vs Rural (Before Inside)	0.43	0.14	1.75
Urban vs Rural (Before Outside)	0.20	0.02	9.92
Urban vs Rural (NTC Inside)	3.39	0.27	178.69
Urban vs Rural (NTC Outside)	**0.10**	**0.02**	**0.45**
Urban vs Rural (ITC Inside)	0.00	0.00	15.19
Urban vs Rural (ITC Outside)	0.47	0.12	2.69

^1^Statistically significant (*p* ≤ 0.05), as bolded, if the HPD interval does not contain 1.

**Table 7:** *Aedes aegypti* abundance and infection rates of homes in clusters with non-treated (NTC) versus insecticide-treated curtains (ITCs).

Area	Inside^1^				Outside^2^			
	NTC		ITC		NTC		ITC	
	#Females (mean)^3^	#inf (rate)^4^	#Females (mean)^3^	#inf (rate)^4^	#Females (mean)^3^	#inf (rate)^4^	#Females (mean)^3^	#inf (rate)^4^
Urban/suburban	1399 (78)	3 (2.1)	1287 (72)	3 (2.3)	1118 (62)	4 (3.6)	1046 (58)	9 (8.6)
Rural	452 (151)	1 (2.2)	206 (69)	0 (0)	198 (66)	6 (30.3)	168 (56)	4 (23.8)

^1^Mosquitoes collected by backpack aspiration inside homes; ^2^Mosquitoes collected by backpack aspiration and BGS traps in the patios of homes; ^3^Number of *Aedes aegypti* females collected (mean number of females per cluster); ^4^Number of DENV infected *Aedes aegypti* collected (DENV Infection rate per thousand mosquitoes).

**Table:** (a) Total number and average of mosquitoes captured per house

Area	Before curtain installation (Baseline)						After curtain installation (Visits 1–9)					
	Total homes^1^		Number of mosquitoes captured inside the house		Average of mosquitoes captured inside the house		Total homes^1^		Number of mosquitoes captured inside the house		Average of mosquitoes captured inside the house	
	NTC	ITC	NTC	ITC	NTC	ITC	NTC	ITC	NTC	ITC	NTC	ITC
Urban	9	5	903	459	100	92	5	2	789	216	158	108
Rural	3	1	335	155	112	155	2	1	623	130	312	130

**Table:** (b) Total number and average of mosquitoes captured per visit

Area	Before curtain installation (Baseline)						After curtain installation (Visits 1–9)					
	Total visits per home^1^		Number of mosquitoes captured inside the house		Average of mosquitoes captured inside the house		Total of visits to homes^1^		Number of mosquitoes captured inside the house		Average of mosquitoes captured inside the house	
	NTC	ITC	NTC	ITC	NTC	ITC	NTC	ITC	NTC	ITC	NTC	ITC
Urban	18	10	903	459	50	46	45	15	789	216	18	14
Rural	4	2	335	155	84	78	18	9	623	130	35	14

^1^More than 50 mosquitoes of all species collected during the study.

**Table 9:** Frequency of Ile1016 in urban/suburban versus rural sites during six consecutive years.

Year	Area	Number of mosquitoes genotyped	Percentage homozygous (Ile/Ile)^1^	Ile allele frequency
2010	Urban/Suburban	450	61.5%	0.80
	Rural	100	7.0%	0.34
2011	Urban/Suburban	1,415	67.4%	0.81
	Rural	67	23.9%	0.50
2012	Urban/Suburban	1,823	57.9%	0.76
	Rural	225	27.6%	0.52
2013	Urban/Suburban	3,391	57.3%	0.74
	Rural	649	37.1%	0.57
2014	Urban/Suburban	1,213	54.3%	0.70
	Rural	424	26.4%	0.45
2015	Urban/Suburban	1,784	70.6%	0.80
	Rural	300	29.0%	0.42

^1^The urban/suburban and rural sites differed significantly (*p* ≤ 0.0001) in the percent of homozygous mosquitoes in each of the 6 years.

**Table 10:** KDR allele frequencies in female and male *Aedes aegypti *mosquitoes collected inside and outside of control (NTC) and intervention (ITC) homes by clusters.

Area	Indoor Collection Visit 1–9						Outdoor Collection Visit 1–9					
	(Backpack collections)						(Backpack and BG trap collections)					
	NTC clusters			ITC clusters			NTC cluster			ITC cluster		
	No. tested	% AA	Freq. of A	No. tested	% AA	Freq. of A	No. tested	% AA	Freq. of A	No. tested	% AA	Freq. of A
Urban/Suburban	1,899	58.08	0.75	1,867	56.40	0.74	2,110	54.12	0.72	2,177	57.23	0.73
Rural	550	33.27	0.54	316	26.90	0.45	444	34.23	0.56	475	21.05	0.40

**Table 11:** Outcome of in-house cylinder bioassay for challenge of insecticide susceptible and resistant strains of *Aedes aegypti* with Olyset Plus and similar but non-treated curtains aged in homes in Mérida City, and amounts of active ingredient (permethrin) and synergist (PBO) present in the samples.

Type of net sample^1^	Location in homes	Months exposed in homes (no. samples)	Mean amount (grams) of active ingredient (permethrin) or synergist (PBO) per kilogram of net sample (% relative to baseline value)		New Orleans insecticide susceptible reference strain of *Ae. aegypti*					Local Mérida City insecticide resistant Vergel strain of *Ae. aegypti*^8^				
					No. tested	Knocked Down (1 hr)		Dead (24 h)		No. tested	Knocked Down (1 hr)		Dead (24 h)	
			Permethrin	PBO		No.	%	No.	%		No.	%	No.	%
Olyset Plus	Baseline^2^	0 (6)	19.1 (100)	8.0 (100)	303	303	100	303	100	298	71	23.8	59	19.8
Olyset Plus	Window^3^	3 (8)^6^	18.6 (97.4)	4.9 (61.2)	412	412	100	412	100	402	93	23.1	49	12.2
Olyset Plus	Window^3^	6 (8)^6^	17.6 (92.1)	3.4 (42.5)	401	399	99.5	399	99.5	402	54	13.4	18	4.5
Olyset Plus	Window^3^	9 (6)^7^	16.5 (86.4)	2.1 (26.2)	304	301	99.0	290	95.4	303	24	7.9	12	4.0
Olyset Plus	Window^3^	11 (6)^7^	16.8 (88.0)	1.6 (20.0)	302	288	95.4	247	81.8	306	14	4.6	7	2.3
Olyset Plus	Interior^4^	3 (5)	18.6 (97.4)	7.1 (88.8)	249	249	100	249	100	252	52	20.6	25	9.9
Olyset Plus	Interior^4^	6 (4)	18.1 (94.8)	6.4 (80.0)	204	204	100	204	100	203	47	23.2	18	8.9
Olyset Plus	Interior^4^	9 (4)	18.2 (95.3)	6.9 (86.2)	202	202	100	202	100	199	43	21.6	23	11.6
Olyset Plus	Interior^4^	11 (4)	17.8 (93.2)	5.5 (68.8)	205	205	100	205	100	206	21	10.2	7	3.4
Non-treated	Baseline^2^	0 (2)	ND	ND	102	0	0	0	0	179	0	0	0	0
Non-treated	Any location^5^	3 (3)	ND	ND	149	0	0	0	0	152	0	0	0	0
Non-treated	Any location^5^	6 (3)	ND	ND	150	0	0	0	0	150	0	0	0	0
Non-treated	Any location^5^	9 (2)	ND	ND	101	0	0	0	0	101	0	0	0	0
Non-treated	Any location^5^	11 (3)	ND	ND	152	0	0	0	0	153	0	0	0	0

^1^Olyset Plus curtain samples were treated with permethrin and PBO; Non-treated curtain samples were similar but without permethrin or PBO. ^2^Baseline curtain samples were not exposed in homes. ^3^Curtain samples continuously exposed in south- or north-facing windows. ^4^Curtain samples placed in the interior part of a home and not exposed to direct sunlight. ^5^Curtain samples exposed in south- or north-facing windows, or in the interior of the home. ^6^Including 4 curtain samples from south-facing windows and 4 from north-facing windows. ^7^Including 3 curtain samples from south-facing windows and 3 from north-facing windows. ^8^Frequency of the knockdown resistance-conferring I1,016 allele mutation of ~90%, with ~80% I1,016 homozygotes, in the F1 generation of the strain; the F2 generation was used in these bioassays. ND = none detected.

**Table 12:** Comparison of backpack aspiration and BGS traps for *Ae. aegypti* and *Cx. quinquefasciatus* mosquito surveillance in patios.

Species	Gender	Total number of mosquitoes in Urban/suburban area		Total number of mosquitoes in Rural area		Total
		Backpack aspiration	BGS trap	Backpack aspiration	BGS trap	
*Aedes aegypti*	female	671	1798	104	404	2977
	male	1262	725	243	305	2535
*Culex quinquefasciatus*	female	920	1547	836	1194	4497
	male	1729	2034	1883	1637	7283
Total		4582	6104	3066	3540	17292

## Data Availability

The raw research data are not available for public use. Readers are welcome to use data presented in this article, when they give appropriate referencing and attributions.

## Supplementary Materials

Supplementary Materials Supplemental Table 1 reports the total number of paired clusters the number of paired intervention and control homes (homes receiving insecticide-treated and non-treated curtains, respectively) and the number of subjects participating in each of the study. The fraccionamientos in which the participating homes were located are also identified. Supplemental Table 2 identifies the species and numbers of mosquitoes captured by backpack aspiration or by BioGents Sentinel traps in and around homes in the urban/suburban and rural study sites. Supplemental Table 3 reports the geometric mean counts for* Aedes aegypti* mosquitoes collected in urban/suburban and rural intervention and control homes by visit and by the number the number of unscreened windows per visit. Supplemental Table 4 reports the geometrical mean counts for all species of mosquitoes collected in urban/suburban and rural intervention and control homes by visit and by the number of unscreened windows for each visit. Supplemental Table 5 provides the results of statistical analyses in three-way contingency tables using the *G*-test of Ile 1,016 homozygote (kdr resistant) frequencies in* Aedes aegypti* mosquitoes collected indoors and outdoors from intervention or control homes (insecticide-treated or non-treated curtains, respectively) in urban/suburban and rural study sites. Supplemental Table 6 reports the mean amounts of permethrin and PBO in insecticide-treated curtains before placement in homes and after exposure in windows or protected in the interior of the homes. Supplemental Figure 1 shows the geographic locations of the urban/suburban and rural study clusters in Yucatán, México.
