# Supplementary material for: The Use of Insecticide-Treated Curtains for Control of Aedes aegypti and Dengue Virus Transmission in “Fraccionamiento” Style Houses in México
Source: J Trop Med. 2018 Jun 19;2018:4054501. doi: 10.1155/2018/4054501 (PMC6029453; doi:10.1155/2018/4054501)

SUPPLEMENTAL TABLES

Supplemental Table 1. Fraccionamiento study sites in urban/suburban and rural areas and the number of homes with either insecticide treated curtains (ITCs) or non-treated curtains (NTCs) and subjects enrolled at the beginning of the study

Area	City (zone)	No. pairs of NTC and ITC clusters / Total no. clusters	No. NTC/ITC homes enrolled at the start	Total no. consented subjects in NTC/ITC homes at the start
**Urban/suburban**	**Mérida (Juan Pablo II)[1]**	9/18	97/88	479/477
	**Mérida – (Vergel)[2]**	3/6	35/36	169/171
	**Mérida – (Sur)[3]**	3/6	35/32	165/139
	**Caucel[4]**	1/2	7/9	41/38
	**Umán[5]**	2/4	20/20	90/76
**Rural**	**Maxcanú[6]**	1/2	11/10	52/38
	**Motul[7]**	2/4	22/23	100/117
**Total**		21/42	227/218	1096/1056

[1] Fraccionamientos in Juan Pablo II: Nora Quintana, Jardines de Yucalpetén,Yucalpetén, Tixcacal Opichén, Juan Pablo II Nora, Bosques del Poniente, Bosques de Mulsay, Villas Tixcacal, Juan Pablo II Ampliación, Juan Pablo II Magisterio, Juan Pablo II Sec. San Pedro, Juan Pablo II Lirios, Juan Pablo II.
[2] Fraccionamientos in Vergel: Vergel I, Vergel II, Vergel III, Vergel IV.
[3] Fraccionamientos in Sur: Unidad Morelos, Brisas de San José, Valle Dorado, Jardines del Sur, Serapio Rendón III, San Nicolás del Sur.
[4] Fraccionamientos in Caucel: Sol Caucel IV, Balcones I.
[5]Fraccionamientos in Umán: ACIM I, ACIM II, Paseos de Itzincab, San Lorenzo
[6]Fraccionamientos in Maxcanú: Puertas del Sol, Villas de Maxcanú.
[7]Fraccionamientos in Motul: Viva, Puertas del Sol, Villas la Hacienda, La Herradura.

Supplemental Table 2. Mosquito species abundance and diversity collected in Urban/suburban and rural sites from fraccionamientos[1]

Species	Sex	Urban/suburban		Rural		Total
		Back pack aspirator	BGS trap	Back pack aspirator	BGS trap	
		Total number of mosquitoes	Total number of mosquitoes	Total number of mosquitoes	Total number of mosquitoes	
Aedes aegypti	female	3,314	1,798	1,161	404	6,677
	male	2,690	725	933	305	4,653
Culex quinquefasciatus	female	2,368	1,547	3,570	1194	8,679
	male	1,634	2,034	4,475	1637	9,780
Ochlerotatus taeniorhynchus	female	413	59	61	1	534
	male	51	7	10	1	69
Ochlerotatus trivittatus	female	17	3	0	10	30
	male	2	0	0	3	5
Aedes bimaculatus	female	1	0	0	0	1
	male	0	0	0	0	0
Aedes cozumelensis	female	0	0	1	0	1
	male	0	0	0	0	0
Anopheles albimanus	female	2	0	0	0	2
	male	0	0	0	0	0
Culex coronator	female	0	0	2	0	2
	male	0	0	0	0	0
Culex nigripalpus	female	6	0	0	0	6
	male	0	0	0	0	0
Ochlerotatus bimaculatus	female	1	0	0	0	1
	male	0	0	0	0	0
Psorophora cyanescens	female	0	0	5	0	5
	male	0	0	0	0	0
Uranotaenia lowii	female	1	0	1	0	2
	male	0	0	0	0	0
Unknown	unknown	1	0	0	0	1
**Total**		10,501	6,173	10,219	3,555	30,448

[1] **Backpack aspirators were used inside and outside of the houses and BGS traps were used outside of the houses.**

Supplemental Table 3. Geometric mean[1] count for all *Aedes aegypti* mosquitoes in urban/suburban and rural areas by visit number, treatment group, and number of unscreened windows in control (NTC) and intervention (ITC) homes

Area	Number of unscreened windows	Treatment	Visit									Overall
			1	2	3	4	5	6	7	8	9	
Urban/suburban	≤ 1	NTC	**1.17**	0.39	0.57	1.23	0.64	**0.68**	0.55	0.28	0.35	0.62
		ITC	**0.42**	0.23	0.51	1.00	0.83	**1.31**	0.87	0.25	0.72	0.65
	2 to 4	NTC	0.77	0.15	0.42	1.00	1.24	0.87	0.58	0.22	0.83	0.64
		ITC	0.83	0.40	0.43	1.23	1.44	1.50	0.84	0.33	0.64	0.80
	≥ 5	NTC	0.79	0.31	0.77	1.87	1.27	1.07	0.99	0.17	1.01	0.86
		ITC	0.78	0.36	0.46	2.23	1.53	0.94	1.48	0.22	0.92	0.90
	Overal	NTC	0.90	0.28	0.58	1.34	1.03	0.87	0.70	0.22	0.71	0.70
		ITC	0.67	0.33	0.47	1.44	1.24	1.23	1.04	0.26	0.76	0.78
Rural	≤ 1	NTC	0.58	0.71	1.09	**3.63**	1.99	1.37	1.33	1.09	**2.61**	1.46
		ITC	0.40	0.19	0.45	**0.34**	0.80	0.57	1.21	0.26	**0.25**	0.47
	2 to 4	NTC	0.53	1.43	0.47	4.47	2.76	2.32	0.94	0.23	4.41	1.56
		ITC	0.06	0.22	1.23	2.21	1.11	1.28	1.17	0.26	1.88	0.92
	≥ 5	NTC	0.66	1.68	0.38	**6.82**	2.82	2.82	1.74	0.44	**6.45**	2.02
		ITC	0.61	0.74	0.51	**1.67**	2.81	0.92	1.57	0.46	**2.06**	1.14
	Overal	NTC	0.59	1.23	0.62	**4.83**	2.51	2.11	1.32	0.55	**4.26**	1.67
		ITC	0.33	0.37	0.69	**1.25**	1.44	0.90	1.31	0.32	**1.22**	0.82

[1]Geometric means are significantly different between the control (NTC) and intervention (ITC) treatment group of homes at the 10% significance level if bolded and at the 5% significance level if bolded and underlined.

Supplemental Table 4. Geometric mean[1] count for all mosquitoes of all species in the urban/suburban and rural areas by visit number, treatment group, and number of unscreened windows in control (NTC) and intervention (ITC) homes

Area	Number of unscreened windows	Treatment	Visit									Overall
			1	2	3	4	5	6	7	8	9	
Urban/suburban	≤ 1	NTC	**1.79**	0.72	0.76	1.58	0.98	1.06	0.82	0.41	0.46	0.90
		ITC	**0.72**	0.32	0.64	1.25	1.24	1.56	1.46	0.51	0.95	0.92
	2 to 4	NTC	1.22	**0.47**	0.58	1.43	1.59	1.26	0.85	0.31	0.91	0.91
		ITC	1.69	**1.16**	0.70	1.52	1.62	1.62	1.44	0.42	1.09	1.21
	≥ 5	NTC	1.74	**1.36**	**1.24**	2.54	1.47	1.71	1.55	0.18	1.09	1.35
		ITC	1.29	**0.76**	**0.69**	2.83	1.94	1.61	1.96	0.52	1.38	1.34
	Overal	NTC	1.57	0.81	0.84	1.81	1.33	1.33	1.05	0.29	0.80	1.04
		ITC	1.20	0.71	0.68	1.79	1.58	1.60	1.61	0.48	1.13	1.15
Rural	≤ 1	NTC	0.85	0.86	1.33	**3.54**	4.09	3.38	3.11	1.45	**3.20**	2.19
		ITC	1.36	0.67	1.09	**0.51**	1.36	2.15	1.44	0.37	**0.38**	0.96
	2 to 4	NTC	1.73	2.29	2.08	5.52	5.28	4.57	0.63	1.25	5.89	2.79
		ITC	1.37	1.47	4.27	3.61	2.63	1.53	2.15	0.32	3.47	2.07
	≥ 5	NTC	3.18	3.28	**1.40**	10.56	3.98	4.36	3.65	0.62	10.42	3.71
		ITC	1.71	2.55	**5.54**	4.51	5.02	2.38	3.60	2.61	4.82	3.45
	Overal	NTC	1.77	1.97	1.58	**6.00**	4.42	4.08	2.15	1.08	**5.91**	2.85
		ITC	1.47	1.44	3.16	**2.37**	2.72	2.00	2.28	0.86	**2.30**	1.99

[1]Geometric means are significantly different between the control (NTC) and intervention (ITC) treatment group of homes at the 10% significance level if bolded and at the 5% significance level if bolded and underlined.

Supplemental Table 5. The effects of clusters, indoor or outdoor NTC vs. ITC, and NTC or ITC indoor vs. outdoor collections on Ile1,016 homozygote frequencies of mosquitoes in urban/suburban and rural areas analyzed in three-way contingency tables using the G-test (Sokal and Rohlf, 1969).

		Urban/Suburban		Rural	
**Indoor NTC vs. ITC**					
Source	*d.f*	*G*	*p-value*	*G*	*p-value*
Cluster x Genotype	15	36.20	0.0017	51.41	<0.0001
Cluster x (NTC vs. ITC)	15	597.24	<0.0001	71.64	<0.0001
Genotype x (NTC vs. ITC)	1	3.70	**0.0544**	3.86	**0.0494**
Cluster x Genotype x (NTC vs. ITC) interaction	17	33.38	0.0101	29.50	0.0302
Cluster x Genotype x (NTC vs. ITC) independence	48	670.52	<0.0001	156.42	<0.0001
**Outdoor NTC vs. ITC**					
Source	*d.f*	*G*	*p-value*	*G*	*p-value*
Cluster x Genotype	15	68.64	<0.0001	33.95	0.0035
Cluster x (NTC vs. ITC)	15	419.77	<0.0001	52.27	<0.0001
Genotype x (NTC vs. ITC)	1	4.20	**0.0403**	20.12	**<0.0001**
Cluster x Genotype x (NTC vs. ITC) interaction	17	45.18	<0.0001	-6.89	NA
Cluster x Genotype x (NTC vs. ITC) independence	48	537.80	<0.0001	99.46	<0.0001
**NTC Outdoor vs. Indoor**					
Source	*d.f*	*G*	*p-value*	*G*	*p-value*
Cluster x Genotype	15	66.03	<0.0001	39.62	0.0005
Cluster x (Indoor vs. Outdoor)	15	412.66	<0.00010	58.14	<0.0001
Genotype x (Indoor vs. Outdoor)	1	6.36	0.0116	0.10	0.7497
Cluster x Genotype x (In vs. Out) interaction	17	31.97	0.0152	11.28	0.8417
Cluster x Genotype x (In vs. Out) independence	48	517.03	<0.0001	109.14	<0.0001
**ITC Outdoor vs. Indoor**					
Source	*d.f*	*G*	*p-value*	*G*	*p-value*
Cluster x Genotype	15	44.90	0.0001	40.93	0.0003
Cluster x (Indoor vs. Outdoor)	15	465.79	<0.0001	65.85	<0.0001
Genotype x ((Indoor vs. Outdoor)	1	2.10	0.1474	3.59	0.0583
Cluster x Genotype x (In vs. Out) interaction	17	40.49	0.0011	16.14	0.5137
Cluster x Genotype x (In vs. Out) independence	48	553.29	<0.0001	126.51	<0.0001

Supplemental Table 6. Mean amounts of active ingredient (permethrin) and synergist (PBO) in insecticide treated curtains at baseline (before placement in homes) and after exposure in windows or in the interior of the homes

		Mean amount of permethrin (g/kg) in ITCs			Mean of amount of PBO (g/kg) in ITCs		
**Months exposed in homes**	**No. curtain samples from windows /interior**	**Baseline**	**Window (% loss)**	**Interior (% loss)**	**Baseline**	**Window (% loss)**	**Interior (% loss)**
3	8 / 5	19.1	18.6 (3)	18.6 (3)	8.0	4.9 (39)	7.1 (11)
6	8 / 4	19.1	17.6 (8)	18.1 (5)	8.0	3.4 (57)	6.4 (20)
9	6 / 4	19.1	16.5 (14)	18.2 (5)	8.0	2.1 (74)	6.9 (14)
11	6 / 4	19.1	16.8 (12)	17.8 (7)	8.0	1.6 (80)	5.5 (31)

Supplemental Figure

Supplemental Figure 1 shows the geographic locations of the urban/suburban and rural study clusters in Yucatán, México.

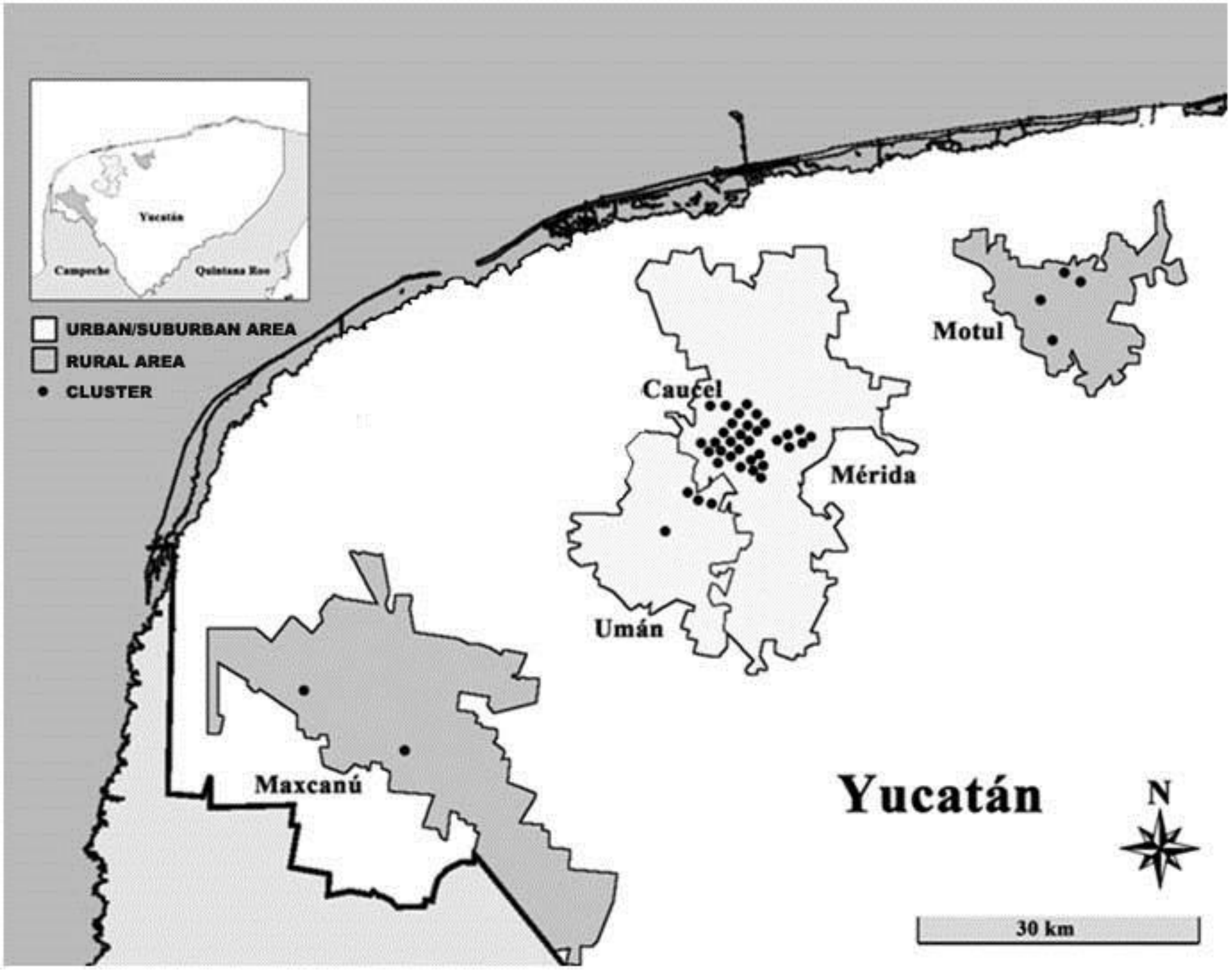

Supplement: Supplementary Materials — Supplemental Table 1 reports the total number of paired clusters the number of paired intervention and control homes (homes receiving insecticide-treated and non-treated curtains, respectively) and the number of subjects participating in each of the study. The fraccionamientos in which the participating homes were located are also identified. Supplemental Table 2 identifies the species and numbers of mosquitoes captured by backpack aspiration or by BioGents Sentinel traps in and around homes in the urban/suburban and rural study sites. Supplemental Table 3 reports the geometric mean counts for Aedes aegypti mosquitoes collected in urban/suburban and rural intervention and control homes by visit and by the number the number of unscreened windows per visit. Supplemental Table 4 reports the geometrical mean counts for all species of mosquitoes collected in urban/suburban and rural intervention and control homes by visit and by the number of unscreened windows for each visit. Supplemental Table 5 provides the results of statistical analyses in three-way contingency tables using the G-test of Ile 1,016 homozygote (kdr resistant) frequencies in Aedes aegypti mosquitoes collected indoors and outdoors from intervention or control homes (insecticide-treated or non-treated curtains, respectively) in urban/suburban and rural study sites. Supplemental Table 6 reports the mean amounts of permethrin and PBO in insecticide-treated curtains before placement in homes and after exposure in windows or protected in the interior of the homes. Supplemental Figure 1 shows the geographic locations of the urban/suburban and rural study clusters in Yucatán, México. [file 4054501.f1.pdf]
